# Intercontinental Movement of Highly Pathogenic Avian Influenza A(H5N1) Clade 2.3.4.4 Virus to the United States, 2021

**DOI:** 10.3201/eid2805.220318

**Published:** 2022-05

**Authors:** Sarah N. Bevins, Susan A. Shriner, James C. Cumbee, Krista E. Dilione, Kelly E. Douglass, Jeremy W. Ellis, Mary Lea Killian, Mia K. Torchetti, Julianna B. Lenoch

**Affiliations:** US Department of Agriculture National Wildlife Research Center, Fort Collins, Colorado, USA (S.N. Bevins, S.A. Shriner);; US Department of Agriculture National Wildlife Disease Program, Fort Collins (K.E. Dilione, J.B. Lenoch);; US Department of Agriculture Wildlife Services, Columbia, South Carolina, USA (J.C. Cumbee Jr);; US Department of Agriculture Wildlife Services, Raleigh, North Carolina, USA (K.E. Douglass);; US Department of Agriculture Veterinary Services, Ames, Iowa, USA (M.L. Killian, M.K. Torchetti)

**Keywords:** influenza, highly pathogenic avian influenza A(H5N1) virus, clade 2.3.4.4, influenza viruses, viruses, intercontinental movement, influenza, respiratory infections, wild birds, dispersal, United States, zoonoses

## Abstract

We detected Eurasian-origin highly pathogenic avian influenza A(H5N1) virus belonging to the Gs/GD lineage, clade 2.3.4.4b, in wild waterfowl in 2 Atlantic coastal states in the United States. Bird banding data showed widespread movement of waterfowl within the Atlantic Flyway and between neighboring flyways and northern breeding grounds.

Influenza A viruses have a worldwide distribution, and wild birds are the primary wild reservoir. Many wild ducks in particular are often repeatedly exposed to and infected with these viruses (hereafter referred to as avian influenza viruses or AIV) with little to no sign of clinical disease (*1*), although highly pathogenic forms of the virus can sometimes cause illness and death in wild birds (*2*). Highly pathogenic lineage viruses identified in 1996 (A/goose/Guangdong/1/1996 [Gs/GD]) have repeatedly spilled over from poultry to wild birds, and eventual emergence of highly pathogenic AIV Gs/GD clade 2.3.4.4 has led to more persistent circulation of these viruses in wild birds and high numbers of illnesses and deaths in poultry on multiple continents (*3*).

One way to better understand AIV movement on the landscape or to identify routes of introduction of novel AIVs is through wild bird band-recovery data (*4*). These data have been collected as part of waterfowl management and conservation efforts in North America since the 1920s (*5*). Spatial locations of where birds are banded and later recovered are recorded and archived, providing data on wild bird movement. For waterfowl, recoveries primarily occur through banded birds being reported as part of hunter harvest activities.

## The Study

Wild bird samples are routinely collected by the US Department of Agriculture, Animal Plant Health Inspection Service, Wildlife Services, National Wildlife Disease Program (National Wildlife Disease Program, US Fish and Wildlife Service permit no. MB124992 0) and screened for AIV in conjunction with the National Animal Health Laboratory Network and with the National Veterinary Services Laboratories (Ames, Iowa, USA) as part of a targeted AIV surveillance program in wild birds (*6*). Samples analyzed in this investigation came from routine wild bird surveillance activities in the US Atlantic Flyway and were primarily obtained from hunter harvest activities, live-trapping, and bird banding operations. These surveillance data, combined with bird band-recovery movement data, can shed light on AIV occurrence on the landscape, and findings in wild birds can act as an early warning system for spillover risk to poultry and humans (*6*).

For these analyses, we initially screened wild bird samples by using an influenza matrix gene real-time, reverse transcription PCR. We then tested matrix gene presumptive positive samples by using H5 and H7 subtype-specific, real-time reverse transcription PCRs. Influenza A virus RNA from wild bird samples was amplified as described (*7*). After amplificationwas completed, we generated cDNA libraries for MiSeq by using the Nextera XT DNA Sample Preparation Kit (Illumina, https://www.illumina.com) and the 500 cycle MiSeq Reagent Kit v2 (Illumina) according to manufacturer instructions. We performed de novo and directed assembly of genome sequences by using IRMA version 0.6.7 (*8*), followed by visual verification in DNAStar SeqMan version 14 (https://www.dnastar.com). For phylogenetic analysis, we downloaded sequences from GISAID (https://www.gisaid.org) and aligned in Geneious 11.1.5 by using MAFFT (https://www.geneious.com), then generated trees by using RAxML (https://cme.h-its.org).

We queried North American Bird Banding Program data (*5*) to find all records from 1960–2021 for 11 dabbling duck species targeted for wild bird surveillance. These species were American black duck (*Anas rubripes*), American green-winged teal (*Anas crecca carolinensis*), American wigeon (*Mareca americana*), blue-winged teal (*Spatula discors*), cinnamon teal (*Spatula cyanoptera*), gadwall (*Mareca strepera*), mallard (*Anas platyrhynchos*), mottled duck (*Anas fulvigula*), northern pintail (*Anas acuta*), northern shoveler (*Spatula clypeata*), and wood duck (*Aix sponsa*). We then limited records for these species to only include birds that were either banded or encountered in North Carolina or South Carolina, USA, and >1 other state or province.

**Figure 1:**
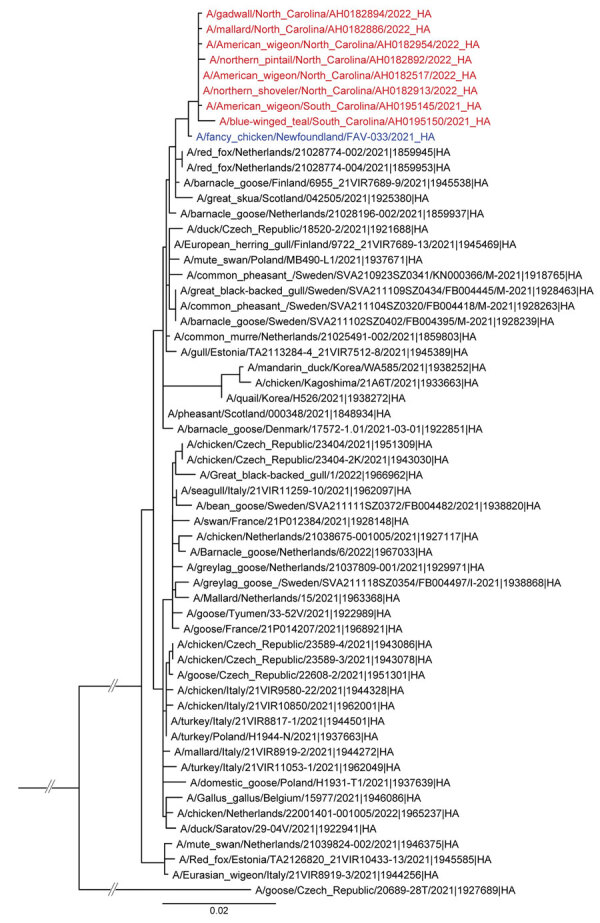
Maximum-likelihood phylogenic analysis of the hemagglutinin gene segment of the first sequenced set of wild bird isolates of highly pathogenic avian influenza A(H5N1) clade 2.3.4.4 virus, United States, 2021. Red indicates US wild bird highly pathogenic detections, and blue indicates closest virus detected in Newfoundland, Canada. MAFFT alignment and RAxML trees were generated in Geneious 11.1.5 (https://www.geneious.com) and visualized in FigTree 1.4.1 (https://tree.bio.ed.ac.uk). Scale bar indicates average nucleotide substitutions per site.

As part of these routine surveillance efforts, we detected Gs/GD lineage clade 2.3.4.4b H5N1 highly pathogenic AIVs in multiple wild birds sampled in North Carolina and South Carolina during December 2021 and January 2022 (Figure 1). Genetic analyses showed that all virus segments were of Eurasian origin (99.7%–99.8% similar; Appendix) and have high identity with December 2021 AIV H5N1 findings in Newfoundland, Canada (Figure 1) (*9*).

A sample was collected on December 30, 2021 from an American wigeon in Colleton County, South Carolina [A/American_wigeon/South_Carolina/AH0195145/2021(H5N1), GISAID accession no. EPI_ISL_9869760]. Immediately after this finding, there was an additional wild bird detection in South Carolina [A/blue-winged_teal/South_Carolina/AH0195150/2021(H5N1), GISAID accession no. EPI_ISL_9876777] and detections in neighboring North Carolina (Figure 1). Another 291 detections in wild birds occurred within 2 months, indicating high susceptibility to infection with a novel virus along with continued transmission and dispersal (Table). All birds were apparently healthy live-trapped or hunter-obtained dabbling ducks (Appendix Table). North American lineage AIV was not found in any of these samples.

**Table:** Detetections of highly pathogenic avian influenza A(H5N1) clade 2.3.4.4 virus in wild birds, United States, December 30, 2021‒March 3, 2022*

State	Wild bird species	No. clade 2.3.4.4 detections
Alabama	American wigeon	1
Connecticut	Mallard	21
	American black duck	9
Delaware	American wigeon	1
	Gadwall	1
	Northern shoveler	5
	American black duck	1
Florida	Blue-winged teal	2
Georgia	American wigeon	1
	Gadwall	1
Kentucky	Gadwall	4
	Mallard	4
Maine	American black duck	6
New Hampshire	Mallard	49
New Jersey	Mallard	21
North Carolina	American green-winged teal	34
	American wigeon	53
	Gadwall	19
	Mallard	14
	Northern pintail	4
	Northern shoveler	8
	Wood duck	3
South Carolina	American wigeon	7
	Blue-winged teal	9
	Gadwall	7
	Northern shoveler	1
Tennessee	Wood duck	2
Virginia	American green-winged teal	2
	Gadwall	1
	Mallard	1
Total detections		292

*All samples collected were in conjunction with the US Department of Agriculture Wildlife Services National Wildlife Disease Program.

**Figure 2:**
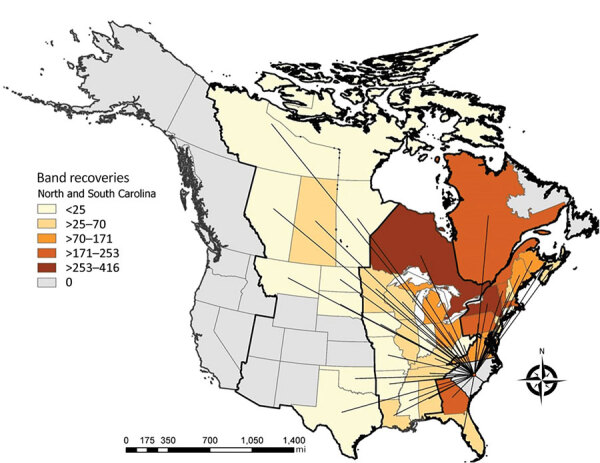
Dabbling duck movements to and from North Carolina and South Carolina, USA, to and from other states or provinces in study of highly pathogenic avian influenza A(H5N1) 2.3.4.4 virus, United States, 2021. Data are based on North American Bird Banding Program data collected during 1960–2021. Color intensities represent number of movements detected between a given state or province and North Carolina or South Carolina. Lines are positioned at the centroid of a given state or province. Bold border lines indicate administrative migratory bird flyways (from west to east: Pacific Flyway, Central Flyway, Mississippi Flyway, and Atlantic Flyway).

Analysis of North American Bird Banding Program data showed broadscale movement of waterfowl throughout North America. Across 11 species of dabbling ducks targeted in surveillance sampling that were historically banded or encountered in North Carolina or South Carolina (and subsequently or previously banded or encountered in another state or province), a total of 64.7% of bird movements were within the Atlantic Flyway, 33.6% of analyzed species were encountered in the Atlantic and the Mississippi Flyways, and 1.7% were encountered in the Atlantic and Central Flyways (Figure 2).

## Conclusions

Although there has been intense focus on intercontinental movement of highly pathogenic AIV from Asia to the North American Pacific Flyway (*10*), viral movement by the trans-Atlantic pathway has been less clear (*11*). Data reported here, in combination with the recent highly pathogenic AIV findings in Newfoundland, Canada (*9*), suggest that wild bird surveillance captured the introduction of a Eurasian-origin highly pathogenic AIV into wild birds by the Atlantic Flyway of the United States. The potential introduction pathway probably includes wild bird migratory routes from northern Europe that overlap Arctic regions of North America and then dispersal farther south into Canada and the United States (*12*).

Band recovery data showed that most dabbling ducks banded in the Atlantic Flyway are also recovered in the Atlantic Flyway, reinforcing the predominance of within flyway movement (*13*). However, data also show routine movement to other flyways, providing a potential mechanism of wider spread dispersal of the virus in North America.

In addition, sequence data indicate that these viruses cluster closely with viruses found in Western Europe during spring of 2021 (Figure 1; Appendix). If viruses were exchanged between North American and Eurasian waterfowl on northern breeding grounds during spring and summer 2021, and then carried south during fall of 2021, these viruses might already be in multiple locations in North America (Figure 2). Because wild bird surveillance has recently been limited to the Atlantic and Pacific Flyways, introductions into the Central or Mississippi Flyways might have gone undetected. Additional detections in wild birds suggest these clade 2.3.4.4b H5 viruses continue to be transmitted (Appendix Table), and further dispersal might be seen once waterfowl migrate to summer breeding areas.

Some findings of highly pathogenic AIVs in wild birds have been associated with repeated spillover of the viruses from domestic birds, which are where mutations to high pathogenicity primarily occur; however, in some cases, Gs/GD lineage viruses now appear to be maintained in wild bird populations (*14*). This potential adaptation of highly pathogenic AIV to wild birds highlights the need for continued wild bird surveillance. In addition, these findings demonstrate that targeted AIV surveillance in wild bird populations can detect newly introduced or emergent AIVs before spillover to domestic poultry. Advanced warnings from wild bird surveillance enable poultry producers to consider altering biosecurity in the face of increased AIV risk and also help inform zoonotic disease potential (*15*).

Appendix Additional information on intercontinental movement of highly pathogenic avian influenza A(H5N1) clade 2.3.4.4 virus to the United States, 2021.
