# Supplementary material for: Intercontinental Movement of Highly Pathogenic Avian Influenza A(H5N1) Clade 2.3.4.4 Virus to the United States, 2021
Source: Emerg Infect Dis. 2022 May;28(5):1006–11. doi: 10.3201/eid2805.220318 (PMC9045435; doi:10.3201/eid2805.220318)
Supplement: Appendix — Additional information on intercontinental movement of highly pathogenic avian influenza A(H5N1) clade 2.3.4.4 virus to the United States, 2021. [file 22-0318-Techapp-s1.pdf]

Article DOI: https://doi.org/10.3201/eid2805.220218

Intercontinental Movement of Highly Pathogenic Avian Influenza A(H5N1) Clade 2.3.4.4 Virus to the United States, 2021

Appendix

Appendix Table. Number and prevalence of clade 2.3.4.4–positive influenza virus detections by species sampled in conjunction with the US Department of Agriculture Wildlife Services National Wildlife Disease Program*

Species	No. positive by species	Clade 2.3.4.4 detections, %
American green-winged teal	36	12
American wigeon	63	22
Blue-winged teal	11	4
Gadwall	33	11
Mallard	110	38
Northern pintail	4	1
Northern shoveler	14	5
Wood duck	5	2
American black duck	16	5
Total	292	100

*Although a variety of wild bird species were sampled, surveillance specifically targeted dabbling duck species.

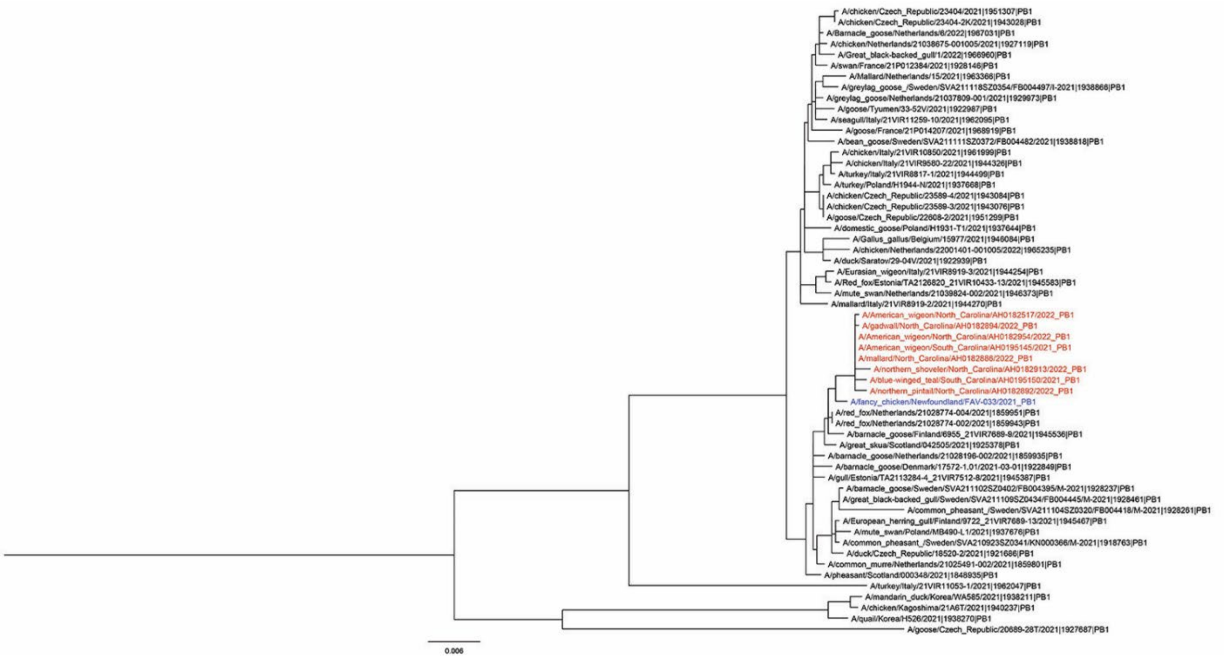

Appendix Figure 1. Maximum-likelihood phylogenic analysis of polymerase basic 1 gene segment. Red indicates outbreak viruses, and blue indicates closest detection in Newfoundland, Canada. Scale bar indicates average nucleotide substitutions per site. MAFFT (https://www.geneious.com) alignment and RAxML (https://cme.h-its.org) trees were generated in Geneious 11.1.5 (https://www.geneious.com), and visualized in FigTree 1.4. (https://tree.bio.ed.ac.uk).

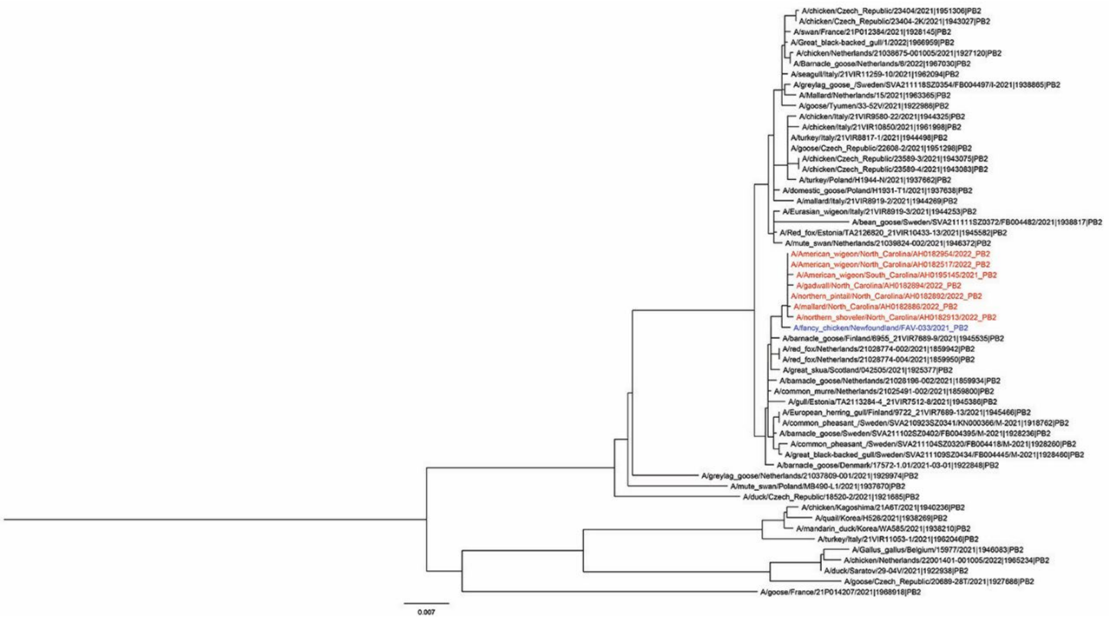

Appendix Figure 2. Maximum-likelihood phylogenic analysis of polymerase basic 2 gene segment. Red indicates outbreak viruses, and blue indicates closest detection in Newfoundland, Canada. Scale bar indicates average nucleotide substitutions per site. MAFFT (https://www.geneious.com) alignment and RAxML (https://cme.h-its.org) trees were generated in Geneious 11.1.5 (https://www.geneious.com), and visualized in FigTree 1.4. (https://tree.bio.ed.ac.uk).

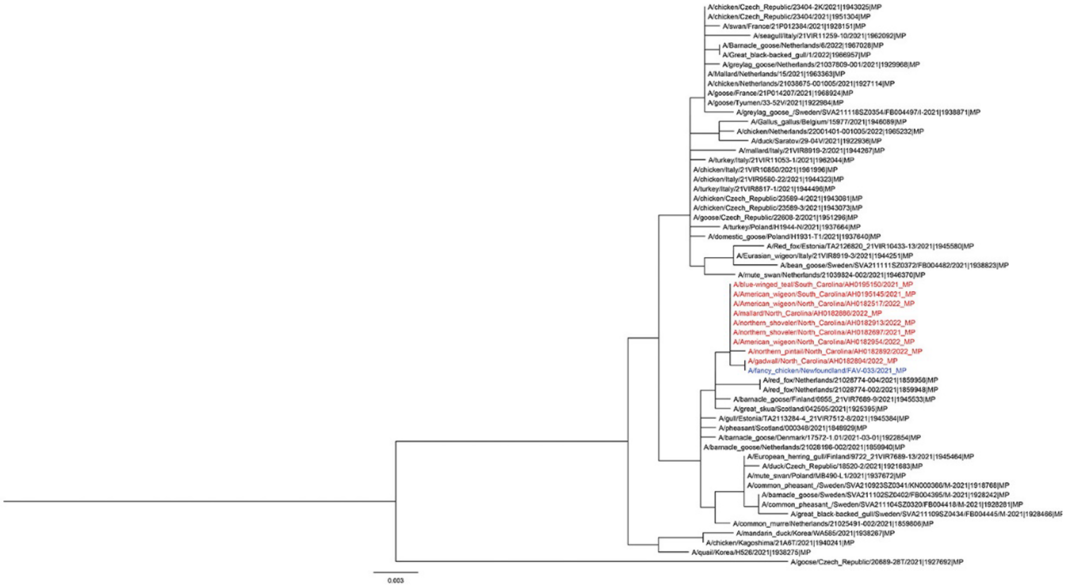

Appendix Figure 3. Maximum-likelihood phylogenic analysis of matrix protein gene segment. Red indicates outbreak viruses, and blue indicates closest detection in Newfoundland, Canada. Scale bar indicates average nucleotide substitutions per site. MAFFT (https://www.geneious.com) alignment and RAxML (https://cme.h-its.org) trees were generated in Geneious 11.1.5 (https://www.geneious.com), and visualized in FigTree 1.4. (https://tree.bio.ed.ac.uk).

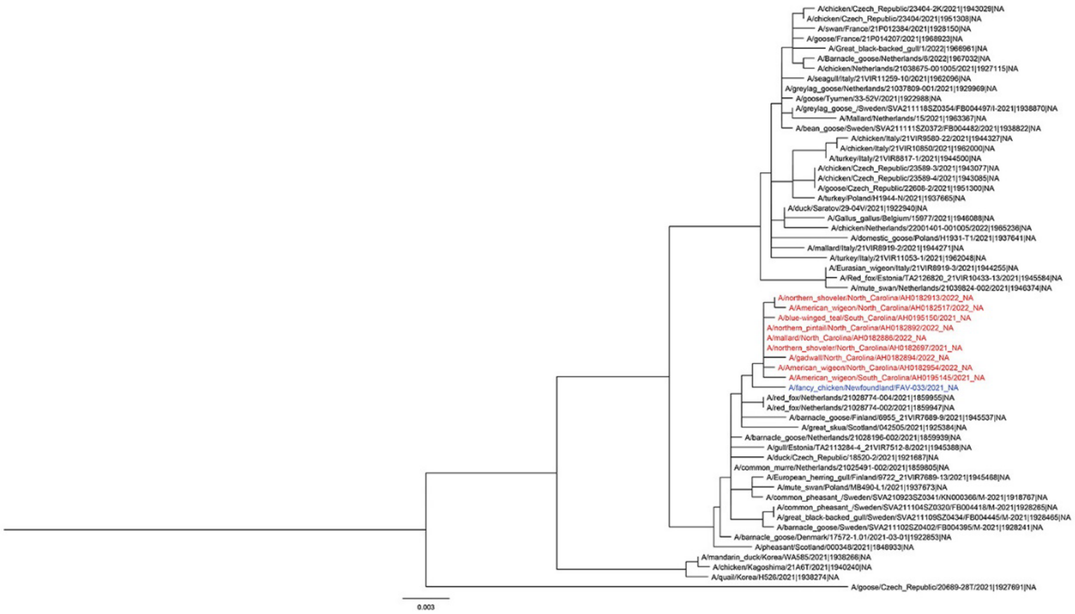

Appendix Figure 4. Maximum-likelihood phylogenic analysis of neuraminidase gene segment. Red indicates outbreak viruses, and blue indicates closest detection in Newfoundland, Canada. Scale bar indicates average nucleotide substitutions per site. MAFFT (https://www.geneious.com) alignment and RAxML (https://cme.h-its.org) trees were generated in Geneious 11.1.5 (https://www.geneious.com), and visualized in FigTree 1.4. (https://tree.bio.ed.ac.uk).

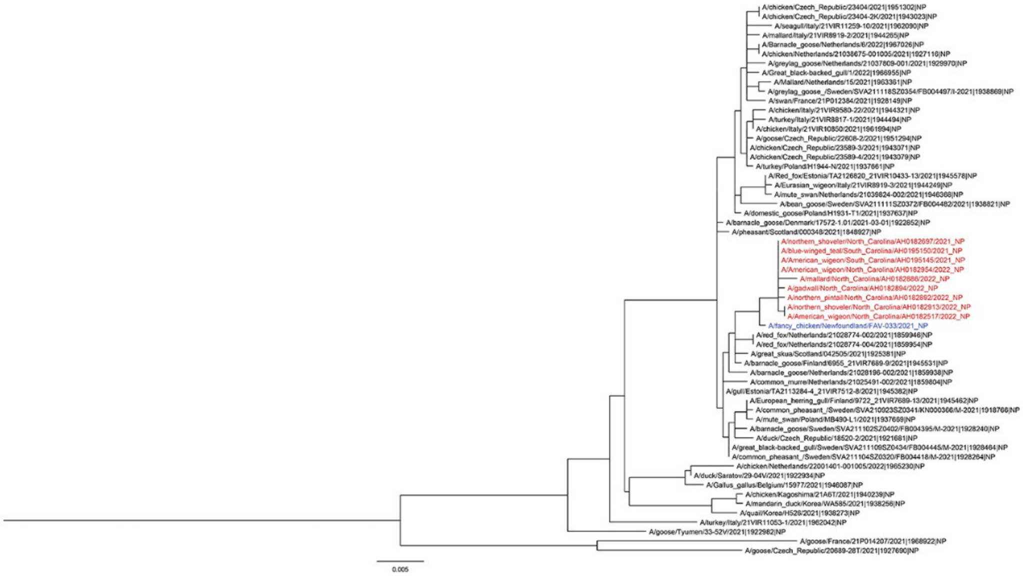

Appendix Figure 5. Maximum-likelihood phylogenic analysis of nucleoprotein gene segment. Red indicates outbreak viruses, and blue indicates closest detection in Newfoundland, Canada. Scale bar indicates average nucleotide substitutions per site. MAFFT (https://www.geneious.com) alignment and RAxML (https://cme.h-its.org) trees were generated in Geneious 11.1.5 (https://www.geneious.com), and visualized in FigTree 1.4. (https://tree.bio.ed.ac.uk).

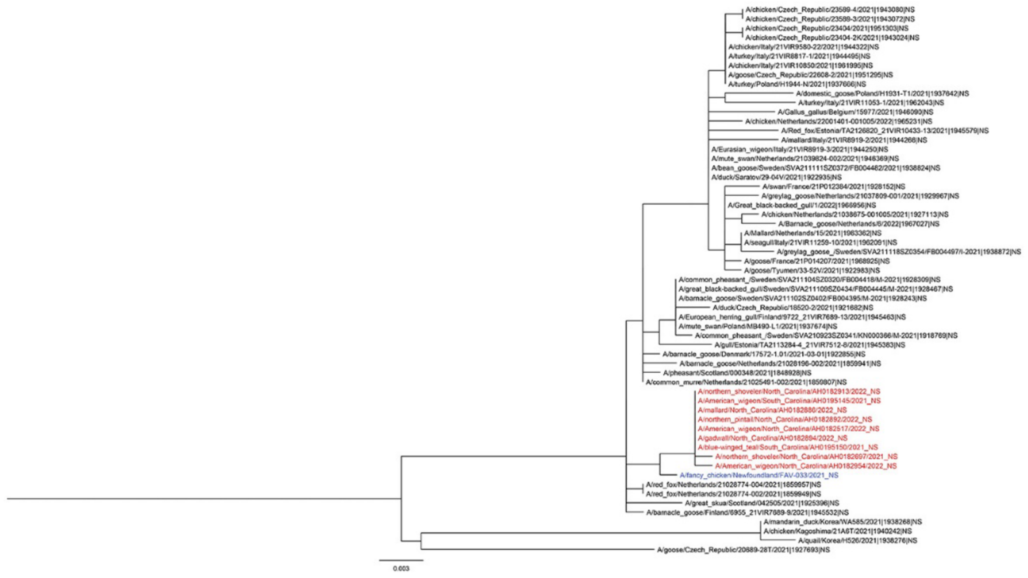

Appendix Figure 6. Maximum-likelihood phylogenic analysis of nonstructural protein gene segment. Red indicates outbreak viruses, and blue indicates closest detection in Newfoundland, Canada. Scale bar indicates average nucleotide substitutions per site. MAFFT (https://www.geneious.com) alignment and RAxML (https://cme.h-its.org) trees were generated in Geneious 11.1.5 (https://www.geneious.com), and visualized in FigTree 1.4. (https://tree.bio.ed.ac.uk).

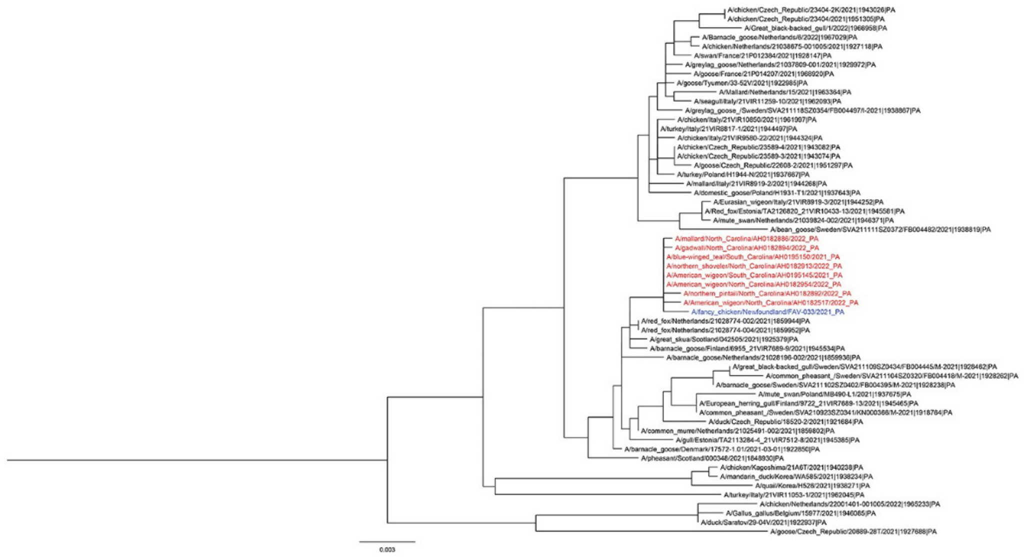

Appendix Figure 7. Maximum-likelihood phylogenic analysis of polymerase acidic gene segment. Red indicates outbreak viruses, and blue indicates closest detection in Newfoundland, Canada. Scale bar indicates average nucleotide substitutions per site. MAFFT (https://www.geneious.com) alignment and RAxML (https://cme.h-its.org) trees were generated in Geneious 11.1.5 (https://www.geneious.com), and visualized in FigTree 1.4. (https://tree.bio.ed.ac.uk).
